# Autopsy-Based Study of Non-accidental Violent Neck Asphyxia in Jordan: A Retrospective Study

**DOI:** 10.7759/cureus.62883

**Published:** 2024-06-22

**Authors:** Shrouq Al-Sabaileh, Mohammad Abusamak, Ali K Al-Buqour, Lama Mehaisen, Radwan Shaher Sabayleh, Talal M Abusamak

**Affiliations:** 1 Department of Forensic Medicine, Al-Balqa Applied University, As-Salt, JOR; 2 Department of Special Surgery, Al-Balqa Applied University, As-Salt, JOR; 3 Department of Ophthalmology, Amman Eye Clinic, Amman, JOR; 4 Department of Forensic Medicine, Ministry of Health Al-Hussein/Salt New Hospital, As-Salt, JOR; 5 Department of Obstetrics and Gynecology, Al-Balqa Applied University, As-Salt, JOR; 6 Department of Internal Medicine, Cairo University, Cairo, EGY; 7 Department of Pharmacology, School of Pharmacy, The University of Jordan, Amman, JOR

**Keywords:** classical signs of asphyxia, autopsy findings of asphyxia, thyroid cartilage fractures, petechial hemorrhages, violent neck asphyxia

## Abstract

Background

There is a global increase in the number of deaths caused by violent neck asphyxia, which includes both suicides and homicides. This research presents autopsy-based findings and analytical demographic data that are pertinent to significant medicolegal cases.

Methods

A retrospective examination of 113 autopsy reports of non-accidental violent neck asphyxia reported to the forensic pathology department of Al-Balqa Governorate in Jordan within the period from January 2010 to March 2023. Age, gender, and autopsy results were documented, along with medicolegal records that included toxicology reports. Conversely, information on the manner of death was derived from police inquest records. For the purpose of this research, all the cases of throttling and ligature strangulation have been determined to be homicidal, and all cases of hanging were classified as suicide.

Results

Males were predominant in hanging (72%), while females were predominant in ligature strangulation (82%). The mean age group in hanging was 21-35 years (51%), while the range for throttling was 36-50 years, and that in strangulation was 2-20 years. Most hanging and throttling cases showed typical external neck findings. Seventy-two percent of hanging cases (n = 68) did not exhibit any internal neck findings, while all throttling cases yielded positive results. Both hanging and strangulation by ligature cases exhibited an absence of thyroid cartilage fracture (83%). Eighty-nine cases (95%) of hanging did not exhibit any injuries to the rest of the body. However, all cases of throttling and ligature strangulation had an almost positive external body injury. In all cases of throttling and ligature strangulation, ocular petechial hemorrhage was present, whereas one-third of the hanging cases had it.

Conclusion

The forensic doctor must observe the pattern of ligature marks and other signs of physical assault found on the neck, meticulously examine the rest of the body, rule out other causes of death, and cooperate with the legal authorities regarding the collection of the history and death scene findings to accurately determine the manner and mechanism of death in cases of violent neck asphyxia.

## Introduction

Cases of violent asphyxia represent a significant portion of the routine responsibilities of any forensic doctor worldwide. This condition results in death when a mechanical obstruction at any level, from the nose to the chest and upper abdomen, creates a state of asphyxiation [1]. However, the high frequency of medicolegal cases related to violent asphyxia via neck obstruction, in particular, necessitates further clinical and investigative concern. This high frequency is mainly related to the global increase in the rates of suicide by hanging [2,3], in addition to the noticeable rise in cases of domestic and sexual violence, in which asphyxia neck violence is considered one of the prime methods of murder [4,5].

From a practical medicolegal perspective, manual strangulation (throttling), ligature strangulation, and hanging constitute the vast majority of methods of violent neck asphyxia [6-8]. The autopsy serves to address crucial legal inquiries regarding the cause and mode of death. Indeed, a trained forensic doctor can easily determine the cause of death in such cases, whether it is vascular or airway occlusion or vagal cardiac arrest [9-11]. This confirmation primarily comes from a professional dissection of the neck and the observation of internal injuries in various neck structures.

However, determining the manner of death, whether homicidal, suicidal, or accidental, remains the critical task facing the forensic doctor. The forensic doctor obtains a proper history, collects circumstantial data, performs an external examination of the neck, examines the pattern of ligature marks, looks for signs of physical neck trauma, meticulously examines the rest of the body, and excludes other causes of death [9,10].

This study’s goal is to investigate cases of non-accidental violent neck asphyxia in terms of demographic factors, the violent methods used, and the number of autopsy findings. This is because these cases are often difficult to handle for both the forensic doctor and the judge because of how complicated the death mechanism and manner of death are.

## Materials and methods

Settings

Medicolegal reports of autopsies of non-accidental violent neck asphyxia were reviewed at the Al-Hussein Al Salt New Hospital in Jordan during the period that extends from January 2010 to March 2023. We included a total of 113 valid cases for analysis.

Medicolegal reports, including toxicology reports, were reviewed, and data including age, gender, and autopsy findings were recorded. Conversely, we collected data on the manner of death from police inquest reports. All cases of hanging were classified as being suicidal, while all cases of throttling and strangulation with ligatures were confirmed as being homicidal in nature.

This study was approved by the institutional board review at Al-Balqa Applied University, As-Salt, Jordan and abides by the tenants of the Helsinki Declaration.

Study type

This was retrospective descriptive observational research that included all the eligible cases of unnatural death due to mechanical obstructions to the neck; cases of drowning, choking, smothering, or being a part of other multiple fatal injuries have been excluded.

Data collection procedures

Multiple variables were extracted from autopsy reports, police inquest records, and death certificates. These variables consist of age, gender, methods of mechanical asphyxia, types of neck compression, and detailed forensic pathological dissection of the neck area and body. Additionally, we documented both external and internal neck injuries. Pertinent external and internal body findings were noted and reported separately.

Data analysis

Data was collected, sorted, and entered into Excel sheets before coding and analysis. Stata SE 14.2 was used to conduct the descriptive and analytical parts of this study [12]. Independent variables, for instance, age and gender, were used to analyze outcome variables such as method of asphyxiation and internal and external neck injuries. X^2^ tests of independence were conducted to find statistically significant associations or differences between the method of asphyxia and other independent and outcome variables. The P-value was calculated for the X^2^ tests with a significance value of <0.05.

## Results

A total of 113 medicolegal autopsies were conducted on deaths of non-accidental violent neck asphyxia during the period from January 2010 to March 2023, out of which 94 (83%) cases were by hanging, 11 (10%) cases were victims of ligature strangulation, and lastly, eight (7%) cases involved manual strangulation. Male victims were 75 (66%), while female victims were 38 (34%).

In Table 1, there was a predominance of males involved in hanging, 68 (72%), while females prevailed in strangulation and throttling, respectively, nine (82%), and three (38%). Moreover, the majority of hanging and strangulation cases fall in the age group of 21-35, while throttling lies mainly within the age group of 36-50. Pearson X^2^ tests were used to compare gender and age to the asphyxiation method.

**Table 1:** Demographic characteristics of the study population (n = 113) P < 0.05 is considered statistically significant.

Variable		Method of death (n = 113)			X^2^	P-value
		Hanging (n = 94, %)	Strangulation (n = 11, %)	Throttling (n = 8, %)		
Gender (n, %)	Male (n = 75)	68 (72%)	2 (18%)	5 (63%)	13	0.002
	Female (n = 38)	26 (28%)	9 (82%)	3 (38%)		
Age (mean ± SD) in years	32.4 (15.0)				103.72	0.277
Age groups	0-20	16 (17%)	6 (55%)	1 (13%)	13.68	0.033
	21-35	48 (51%)	2 (18%)	2 (25%)		
	36-50	18 (19%)	2 (18%)	4 (50%)		
	>51	12 (13%)	1 (9%)	1 (13%)		

The authors conducted Pearson’s chi-squared tests in Table 2 to investigate the significance of differences in the characteristics of pathological findings in violent asphyxia fatalities.

**Table 2:** Autopsy findings with different methods of violent asphyxiation (n = 113) P < 0.05 is considered statistically significant.

Variables		Method of death (n = 113)			Total n	X^2^	P-value
		Hanging (n = 94, %)	Strangulation (n = 11, %)	Throttling (n = 8, %)			
Internal neck injuries							
Neck: muscle contusions, hyoid bone fractures, cricoid cartilage fracture ecchymosis of the larynx, and thyroid gland contusions	Absent	68 (72%)	2(12%)	0 (0%)	70	29.112	<0.001
	Present	26 (28%)	9 (82%)	8 (100%)	43		
Thyroid cartilage fracture	Absent	78 (83%)	9 (82%)	3 (38%)	90	9.44	0.009
	Present	16 (17%)	2 (18.18)	5 (63%)	23		
External neck injuries/mark pattern	No mark	0 (0%)	1 (33.3)	2(25%)	3	40.818	<0.001
	Typical mark	89 (95%)	5 (46%)	6 (75%)	100		
	Atypical mark	5 (5%)	5 (46%)	0	10		
Body injuries	Absent	89 (95%)	1 (9%)	0(0%)	90	78.189	<0.001
	Present	5 (5%)	10 (91%)	8 (100%)	23		

Internal neck injuries

There were no internal neck injuries in almost two-thirds of hanging cases (neck muscle contusions, hyoid bone fractures, cricoid cartilage fracture ecchymosis of the larynx, or thyroid gland contusions). However, all cases of throttling and most cases of ligature strangulation had internal neck findings (100% and 82%), respectively.

Thyroid cartilage fracture

The majority of hanging victims and ligature strangulations had no fracture. In contrast, 63% of throttling cases yielded positive results.

External neck injuries

The majority of hanging cases demonstrated a typical pattern of ligature marks on the neck, which was oblique, incomplete, and above the thyroid cartilage (95%). Counter-ligature marks were present in cases of ligature strangulation, with nearly half of the cases exhibiting the typical mark (transverse, complete, and at or below the thyroid cartilage), while the other half (five cases) exhibited atypical marking (in which the aforementioned characteristics are not necessarily present or presented in a reversed pattern). In throttling, six out of eight cases exhibited the typical findings of throttling (fingernail abrasions or bruises), while the other two cases had no external neck injury.

External body injuries

Ninety-five percent of hanging cases showed no injuries to the rest of the body, while in all cases of throttling and ligature strangulation, there were insults to the body (100%), respectively.

Table 3 shows the method applied in relation to the occurrence of ocular petechial hemorrhage as follows: all cases of throttling and ligature strangulation demonstrated ocular petechial hemorrhage, while only one-third of the cases of hanging showed their presence.

**Table 3:** Ocular hemorrhage vs. method of death (n = 113)

Variables		Method of death (n = 113)			X^2^	P-value
		Hanging (n = 94, %)	Strangulation (n = 11, %)	Throttling (n = 8, %)		
Ocular (sclera/conjunctiva) hemorrhage	Absent	66 (70%)	0	0	32.07	<0.001
	Present	28 (30%)	11 (100%)	8 (100%)		

## Discussion

There has been a noticeable global rise in violence rates across all categories [13,14], with a notable surge in deaths from violent neck asphyxia, leading to a rise in hanging suicide rates as a preferred method [2,3]. Additionally, homicidal neck violence, specifically ligature strangulation and throttling, has become more prevalent, primarily in cases of domestic violence and sexual assaults [6-8]. As a result, the burden of properly diagnosing these cases and determining the manner in which they died is becoming increasingly important for both the forensic doctor and the jurist.

In this study, hanging was found to be the most common method of violent neck asphyxia, followed by ligature strangulation, and lastly, throttling (83%, 10%, and 7%), respectively. Males prevail in suicidal hanging (72%); females account for only 28%; and females are the main victims in homicidal asphyxia cases (63%) in relation to male victims (37%). Our results were in consensus with other previous studies [6-8,15].

The average age of the male victims was 34 years, while the female victims were 29.2 years old. Two female children, ages two and nine, suffered from strangulation during a sexual assault, which resulted in their deaths. Meanwhile, an 80-year-old man, the eldest victim of strangulation, lost his life to a relative during a domestic dispute. Suicidal hanging cases also included two children: a nine-year-old male and a seven-year-old female who took their own lives due to a video game challenge. In addition, toxicological analyses were performed in close to all of the cases, and only two out of 113 victims tested positive for blood alcohol, who were females murdered by ligature strangulation.

The meticulous external clinical examination is as vital as the internal findings revealed after dissection. Inspection of the whole region of the front, back, and sides - not forgetting skin folds in old and obese bodies - all are significant in approaching a proper mechanism and manner of death [10].

The majority of cases showed a regular pattern of incomplete and oblique ligature marks hanging above the thyroid cartilage. Since all cases were suicidal in nature, they typically utilized the typical high points of suspension either indoors or outdoors, such as trees and pillars. Only 5% displayed an atypical pattern, a phenomenon often observed in suicide cases under police custody or in mental asylums where high points of suspension and traditional ligatures are accessible [16,17].

However, five out of 11 cases of strangulation had an atypical pattern of ligature marks on the skin, not being below or at the level of the thyroid cartilage and neither horizontal nor continuous around the neck circumference. This can be explained by the fact that all these cases were female victims of assault by male attackers, with disproportionate physical strength and height in most cases. Two-thirds of the throttling cases showed fingernail abrasions and bruises, as elicited in other studies [18,19]. One case of strangulation and two cases of throttling showed no evidence of violence on their necks; all had a fabric barrier on their skin during the attack (high collar, scarves, and keffiyeh).

As expected by the aggressive homicidal nature of death in throttling and ligature strangulation, all cases showed external injuries on body parts other than the neck, most of which were bruising, scrapings, first- and second-degree burns, and, to a lesser extent, superficial cuts in a few cases. The injuries primarily affected the face, limbs, breasts, and genitalia. Being suicidal, only 5% of hanging demonstrated injuries to the rest of the body. In two male cases, these injuries were explained in the context of complex suicide, where multiple methods of killing one’s self are used, which were in our cases hesitated or superficial wrist cutting, while the remaining three cases had trivial abrasions on limbs of friction nature.

Internal neck findings include neck muscle contusions, hyoid bone fractures, cricoid cartilage fractures, larynx ecchymosis, and thyroid gland contusions. Seventy-two percent of hanging autopsies revealed no findings, with the exception of a minor subcutaneous contusion beneath the ligature mark. Conversely, all throttling cases showed 100% internal findings, which aligned with the findings of previous studies. Nevertheless, the rapidity of their demise due to vagal cardiac arrest explains the absence of findings in two out of 11 cases of strangulation (18%), both involving female children aged two and nine years [11,20].

Thyroid cartilage was intact in 83% of hanging and 82% of strangulation, respectively. This could be linked to the young study sample; the mean age of males is 34 and that of females is 29 [21,22]. However, 17% of hanging cases had fractures, most of which were in the cartilage's superior horns rather than the body’s inferior horns. This may be attributed to the superior traction of the highly located ligature applied to the neck [23,24].

Petechial hemorrhage, one of the cardinal signs of asphyxia [9], was studied regarding its appearance in the conjunctiva and sclera. All cases of violent homicidal asphyxia have a positive occurrence rate of 100%, while 70% of hanging cases demonstrated the absence of petechiae [23,24]. This disparity in the appearance of petechial hemorrhage between the three common types of violent neck asphyxia in favor of strangulation and throttling would emphasize the postulated vascular mechanism over that of the hypoxic endothelial injury mechanism of their occurrence. The beating heart maintains persistent arterial blood flow against the obstructed venous flow that has been occluded via violence applied to the neck, leading to rupturing small venules and hence the formation of such hemorrhages. Accordingly, the absence of petechiae in the majority of hanging cases could be attributed to the swift cessation of cardiac activity due to vagal inhibition, the cardiac arrest mechanism of death [10,11].

Limitations in this study may include the small sample of ligature strangulation and throttling in comparison to hanging cases.

## Conclusions

The present study found that results regarding demographics, external examination, and autopsy findings were in consensus with other studies conducted in other countries. All cases of ligature strangulation and throttling showed positive findings of ocular petechial hemorrhages, but their absence in most suicidal hanging cases leads us to believe that the obstructive vascular theory, rather than hypoxic endothelial injury, explains their formation mechanism. Accordingly, their absence also strengthens the explanation of the mechanism of rapid death in many cases of hanging as being due to the vagal cardiac arrest accompanying many cases of suicidal hanging.

The forensic doctor must observe the pattern of ligature marks and other signs of physical assault found on the neck, meticulously examine the rest of the body, rule out other causes of death, and cooperate with the legal authorities regarding the history and death scene findings in order to properly determine the manner of death.
